# Supplementary material for: Tead1a Initiates Transcriptional Priming Through the TEAD1a/YAP‐Notch1‐Spi1/Cebpα Axis to Promote Neutrophil Fate
Source: Adv Sci (Weinh). 2025 Aug 28;12(41):e05441. doi: 10.1002/advs.202505441 (PMC12591127; doi:10.1002/advs.202505441)
Supplement: Supplementary file 4 — Supplementary Dataset [file ADVS-12-e05441-s004.zip › 3-4.zip!/3-4/02-4.cluster/cluster9/Enrich/ego_BP.pdf]

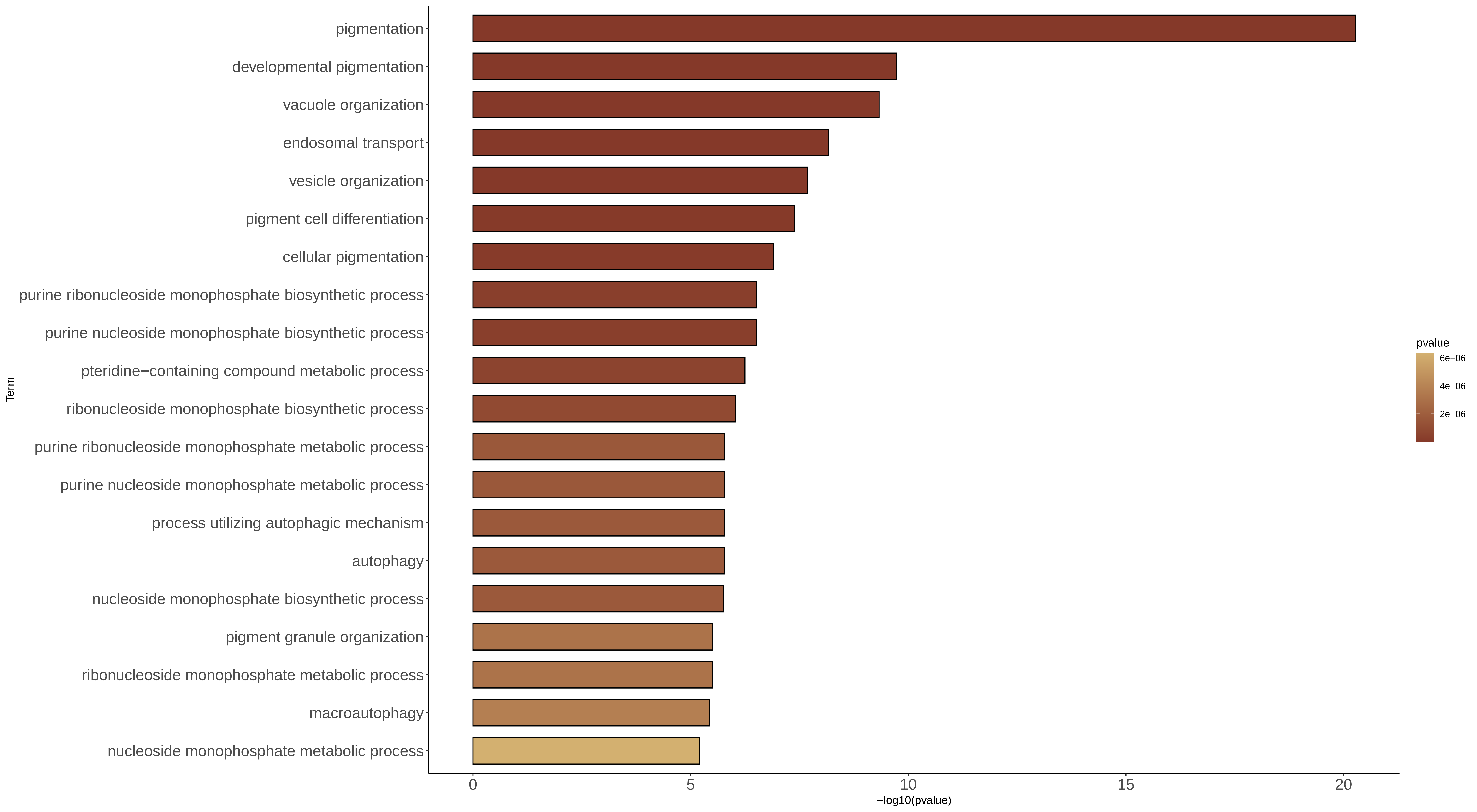

pigmentation
developmental pigmentation
vacuole organization
endosomal transport
vesicle organization
pigment cell differentiation
cellular pigmentation
purine ribonucleoside monophosphate biosynthetic process
purine nucleoside monophosphate biosynthetic process
pteridine−containing compound metabolic process
ribonucleoside monophosphate biosynthetic process
purine ribonucleoside monophosphate metabolic process
purine nucleoside monophosphate metabolic process
process utilizing autophagic mechanism
autophagy
nucleoside monophosphate biosynthetic process
pigment granule organization
ribonucleoside monophosphate metabolic process
macroautophagy
nucleoside monophosphate metabolic process
Term
0
5
10
15
20
−log10(pvalue)
pvalue
6e−06
4e−06
2e−06
